# Hostility in medication-resistant major depression and comorbid generalized anxiety disorder is related to increased hippocampal–amygdala 5-HT_2A_ receptor density

**DOI:** 10.1007/s00406-021-01243-1

**Published:** 2021-04-27

**Authors:** Chris Baeken, Yanfeng Xu, Guo-Rong Wu, Robrecht Dockx, Kathelijne Peremans, Rudi De Raedt

**Affiliations:** 1 Department of Psychiatry and Medical Psychology, Ghent Experimental Psychiatry (GHEP) Lab, Ghent University, Ghent, Belgium; 2 Department of Psychiatry, Vrije Universiteit Brussel (VUB), Universitair Ziekenhuis Brussel (UZBrussel), Laarbeeklaan 101, 1090 Brussels, Belgium; 3 Department of Electrical Engineering, Eindhoven University of Technology, Eindhoven, The Netherlands; 4 Department of Veterinary Medical Imaging and Small Animal Orthopaedics, Faculty of Veterinary Medicine, Ghent University, Merelbeke, Belgium; 5 Key Laboratory of Cognition and Personality, Faculty of Psychology, Southwest University, Chongqing, China; 6 Department of Nutrition, Genetics and Ethology, Faculty of Veterinary Medicine, Ghent University, Merelbeke, Belgium; 7 Department of Experimental Clinical and Health Psychology, Ghent University, Ghent, Belgium

**Keywords:** Major depressive disorder, Generalized anxiety disorder, Comorbidity, R91150 SPECT, serotonin 5-HT2A receptor

## Abstract

Major depressive disorder (MDD) and generalized anxiety disorder (GAD) are severe and difficult-to-treat psychiatric illnesses with high rates of comorbidity. Although both disorders are treated with serotonergic based psychotropic agents, little is known on the influence of the serotonergic neurotransmitter system on the occurrence of comorbid GAD when clinically depressed. To investigate this poorly understood clinical question, we examined the involvement of frontolimbic post-synaptic 5-HT_2A_ receptors in 20 medication-resistant depressed (MRD) patients with half of them diagnosed with comorbid GAD with ^123^I-5-I-R91150 SPECT. To explore whether 5-HT_2A_ receptor-binding indices (BI) associated with comorbid GAD could be related to distinct psychopathological symptoms, all were assessed with the symptom Checklist-90-Revised (SCL-90-R). MRD patients with comorbid GAD displayed significantly higher 5-HT_2A_ receptor BI in the hippocampal–amygdala complex, compared to MRD patients without GAD. Correlation analyses revealed that the 5-HT_2A_ receptor BI in these areas were significantly related to the SCL-90-R subscale hostility (HOS), especially for those MRD patients with comorbid GAD. Comorbid MRD-GAD may be characterized with increased hippocampal–amygdala 5-HT_2A_ receptor BI which could represent enhanced levels in hostility in such kinds of patients. Adapted psychotherapeutic interventions may be warranted.

## Introduction

Major depressive disorder (MDD) and generalized anxiety disorder (GAD) are widespread mental health problems and both illnesses are associated with high recurrence, comorbidity, and they prove to be difficult to treat, so treatment resistance is not uncommon [1, 2]. Pharmacologically managed very similarly with serotonergic based antidepressants [3], it remains, however, unclear how the serotonergic system is involved in the comorbid occurrence of GAD in the depressed state. Better neurobiological insights on the presence of comorbid GAD in MDD could be of considerable interest to fine-tune treatment algorithms in this difficult-to-treat group and certainly in the medication-resistant state.

One possible target for investigation could be the G-protein coupled post-synaptic 5-HT_2_ receptor, which have been found to be involved in the pathophysiology of mood and anxiety disorders, suicide ideation, and MDD treatment (non)response [4, 5]. Animal and human studies have shown that the 5-HT_2A_ receptor is implicated in the context of anxiety-related behaviors and MDD (see review by [6]). In addition, 5-HT_2A_ receptor dysfunctions of frontolimbic processes are often observed in both GAD [7, 8] and MDD patients [9]. Indeed, functional brain-imaging studies in MDD, as well as in GAD, have also demonstrated deregulated neuronal processing in the frontolimbic areas resulting in enhanced stress sensitivity disturbed emotion regulation processes, rumination, and worrying [10–12].

Recently, more attention has been given to the involvement of ‘hostility’ and ‘anger’ in the depressed state [13, 14], which refers to an emotional state varying from mild annoyance to rage [15]. Moreover, elevated levels of multiple dimensions of anger characterize individuals who meet diagnostic criteria for GAD [16]. Here intolerance of uncertainty could be a critical construct underlying the pathophysiology of GAD, and maladaptive behavioral and cognitive reactivity (e.g., biased contextual interpretations of the situation, poor decision-making) may not only increase worry and anxiety [17], but intolerance of uncertainty may also mediate the relationship between GAD symptoms and anger [18]. There is some evidence that supports positive cortical 5-HT_2A_ receptor involvement in aggression, anger, and impulsivity [19]. Surprisingly, no studies have investigated serotonergic differences of comorbid GAD in the treatment-resistant depressed (MRD) state.

Consequently, we collected 20 antidepressant-free (unipolar) melancholic MRD patients (10 with current comorbid GAD) documented to be resistant to several pharmacological interventions, and to examine the influence of the 5-HT_2A_ receptor, we used SPECT and 4-amino-*N*-[1-[3-(4-fluorophenoxy) propyl]-4-methyl-4-piperidinyl]-5-iodo-2-methoxybenzamide (^123^I-5-I-R91150) as the radioligand [20] focusing on the frontolimbic areas. We also assessed the symptom Checklist-90-R (SCL-90-R [21]) to further investigate the relationship between SCL-90-R subscales (including a range of psychological measures, besides depression and anxiety, cognitive and conative symptoms such as hostility) and the 5-HT_2A_ receptor BI, to enlarge our insights on the nature of the serotonergic involvement in the co-occurrence of GAD in patients diagnosed with MRD.

Although primary diagnosis was MRD for all patients, we hypothesized that MRD patients with comorbid GAD would display increased frontolimbic 5-HT_2A_ binding BI when compared to those without the GAD comorbidity. Although we explored whether any of the SCL-90-R subscale scores could be related to distinct 5-HT_2A_ receptor BI between MRD patients with or without comorbid GAD, we expected that higher anxiety and the hostility subscale scores would be related to frontolimbic 5-HT_2A_ binding BI.

## Methods

### Participants

In accordance with the guidelines laid down in the declaration of Helsinki (2004) this study was approved by the Ethical Board of the University Hospital of the Vrije Universiteit Brussel. All patients gave informed consent to participate in the study and for publication. Twenty MRD patients (female: male = 13:7; age = 45.70 years, SD = 9.52) were included using the Dutch version of the Mini-International Neuropsychiatric Interview (M.I.N.I., edition 5.0.0 based on DSM-IV [22]), and all were diagnosed with unipolar melancholic MDD. Depression severity was assessed with the Beck Depression Inventory (BDI-II-NL [23]). Ten patients (50% of the sample) were diagnosed with comorbid GAD. Comorbid psychosis, bipolarity, and substance abuse/dependence were exclusion criteria. Since increased 5-HT2_(A)_ receptor BI are reported in suicide victims [4], suicidal attempts during the current depressive episode were considered as an exclusion criterion. According to the Thase and Rush criteria [24], patients were considered at least stage III treatment resistant: all had at least a minimum of two unsuccessful treatment trials with SSRI/NSRI and one failed clinical trial with a TCA. After a washout period, all patients were free from antidepressant, neuroleptics and mood stabilizers for at least two weeks before scanning. Only benzodiazepines taken on steady dose were allowed. This study was part of a larger investigation examining brain 5-HT_2A_ receptor BI in medication-resistant depression. Given our recent focus on comorbidity in treatment-resistant depression and GAD [25], we re-examined our initial cohort of 21 MRD patients used in an rTMS treatment study [26]. For this study, only the baseline scans were included, before patients underwent the rTMS treatment. Detected retrospectively, one male patient (38 years) diagnosed with MRD and comorbid GAD had an additional opioid addiction (paracetamol codeine for diffuse pain symptoms). Since opioids can increase intrasynaptic levels of serotonin [27], this patient was not included in the current study, nor in another study examining the characterization of 5-HT2A receptor BI in MRD compared to age- and gender-matched drug-naive first episode MDD patients [28].

### SCL-90-R

All patients were assessed with the Dutch version of the Symptom Checklist-90-R (SCL-90-R [21]). This 90-item self-report symptom inventory is designed to reflect psychological symptom patterns of psychiatric and medical patients [29]. On a five-point Likert scale, participants rate the degree to which they have experienced symptoms during the past week. The symptom dimensions in the Dutch version are labeled as somatization (SOM), insufficiency in thought and behavior (IN), interpersonal sensitivity (SEN), depression (DEP), anxiety (ANX), hostility (HOS), agoraphobia (AGO), sleeping problems (SLA), and rest symptoms not otherwise specified (REST). The mean value of all separate items is referred to as the Global Severity Index (GSI), an estimate of general psychological distress in psychiatric populations. Given that we were primarily interested in the first eight SCL-90-R subscales (and not the non-specified rest category), we only focused on SOM, IN, SEN, DEP, ANX, HOS, AGO, and SLA.

### SPECT brain imaging

Static SPECT-imaging was performed with a Siemens MultiSPECT triple-headed gamma camera, equipped with parallel-hole medium-energy collimators. For thyroid blockage all patients received oral Lugol’s solution containing 400 mg of potassium iodide 15 min prior to injection of an average dose of 150 MBq ^123^I-5-I-R91150. SPECT acquisition was performed at a minimum of 120 min after administration of the tracer. Data were collected from 96 angular positions over 360° in a 128 × 128 matrix, with a total acquisition time of 32 min. Reconstruction of the acquired projection images was performed using an iterative reconstruction algorithm (Ordered Subset Expectation Maximization, OSEM, 8 iterations, 8 subsets) and filtered with a 3D Gaussian using 15 mm full width at half-maximum.

SPECT scans were automatically co-registered to a template image placed in a predefined stereotactic (image) space (BRASS; Nuclear Diagnostics Ltd., Sweden) (See also [30]). The sequential acquisition of transmission and emission images was further used to anatomically standardize the emission image using the same linear parameters as those used for the transmission image [31]. All images were visually double-checked to ensure correct anatomical positioning of the predefined VOIs [32].

Radioactivity estimates in the volumes of interest (VOI) were assumed to represent total ligand binding (specific plus nonspecific binding plus free ligand) [32]. Since very few 5-HT_2A_ receptors are present in the cerebellum, this region was chosen to represent nonspecific activity [20]. Calculation of relative indices of specific BI was performed by VOI normalization to the activity per volume element in the cerebellum. Under these pseudo-equilibrium circumstances, BI is directly related to the in vivo receptor density (Bmax) and affinity (Kd). BI was defined as (target activity-background activity in the brain)/(background activity) which was operationally estimated as (counts/pixel in VOI-counts/pixel in the cerebellum)/(counts/pixel in the cerebellum) [31].

Given the relatively small study sample, for the analysis we limited our number of VOIs to six: the brainstem, the dorsolateral prefrontal cortex (left + right DLPFC), orbitofrontal cortex (left + right OFC), ventrolateral prefrontal cortex (left + right VLPFC), anterior cingulate cortex (left + right ACC), hippocampal–amygdala complex (left + right HippAM).

### Statistical methods

All statistical analyses were performed with SPSS 25 (Statistical Package for the Social Sciences, IBM, Chicago, USA). Where necessary, we applied the Greenhouse–Geisser correction to ensure the assumption of sphericity. The significance level was set at *p* < 0.05, two-tailed, except for the SCL-90-R. Since the SCL-90-R was introduced for exploratory purposes and given that we were primarily interested in the first eight subscales, labeled SOM, IN, SEN, DEP, ANX, HOS, AGO, and SLA, for these eight subscale analyses with VOI 5-HT_2A_ receptor-binding indices, here the *p* value, two-tailed was Bonferroni corrected for the number of significant VOIs derived and eight subscales, was set at *p* < 0.00625, two-tailed (Bonferroni correction).

#### Behavioral analysis

Concerning the behavioral outcome results, we compared demographics and SCL-90-R questionnaire scores between MRD patients with and without comorbid GAD with *X*^2^ or *t* tests. See for details Table 1.

**Table 1:** Demographics

	All MRD patients	Without GAD	With GAD	X^2^ or T tests	p value
Gender (F:M)	13:7	6:4	7:3	0.22	0.64
Age	45.70 (9.52)	49.40 (10.41)	42.00 (7.26)	1.85	0.08
BDI-II	33.30 (11.17)	32.00 (11.95)	34.60 (10.80)	0.51	0.62
Depression episode duration (years)	4.38 (3.49)	3.35 (2.00)	5.40 (4.41)	1.34	0.20
Benzodiazepines (mg/day)	12.30 (23.41)	16.00 (28.75)	8.60 (17.31)	0.70	0.50
Suicide risk (MINI)*	1.00 (2.00)	0.50 (1.25)	1.00 (1.25)	2.10	0.18
SCL-90					
Subscales					
ANX	28.68 (7.54)	26.60 (6.29)	31.00 (8.47)	1.29	0.21
AGO	16.47 (7.19)	13.80 (4.16)	19.44 (8.83)	1.75	0.11
DEP	58.21 (13.46)	54.10 (14.72)	62.78 (10.93)	1.45	0.18
SOM	33.74 (9.13)	31.30 (8.10)	36.44 (9.91)	1.24	0.23
IN	28.84 (7.49)	24.80 (4.02)	33.33 (8.06)	2.97	< 0.01
SEN	44.47 (14.91)	39.80 (12.48)	49.67 (16.35)	1.49	0.16
HOS	11.42 (4.30)	9.50 (2.17)	13.56 (5.15)	2.09	0.05
SLA	9.58 (3.90)	9.10 (3.45)	10.11 (4.48)	0.56	0.59
REST	20.74 (4.93)	19.40 (4.88)	22.22 (4.82)	1.27	0.22
GSI	252.16 (59.91)	228.40 (46.86)	278.56 (64.15)	1.96	0.07

Data are presented as ratio, means and standard deviations

*MDD* major depressive disorder, *GAD* generalized anxiety disorder, *BDI–II* 21-item Beck Depression Inventory, *F* female, *M* male. Significance set at *p* < 0.05, two-tailed. Somatization (SOM), insufficiency in thought and behavior (IN), interpersonal sensitivity (SEN), depression (DEP), anxiety (ANX), hostility (HOS), agoraphobia (AGO), sleeping problems (SLA), and rest symptoms not otherwise specified (REST), Global Severity Index (GSI)

*In medians and interquartile ranges

#### SPECT analysis

**Fig. 1:**
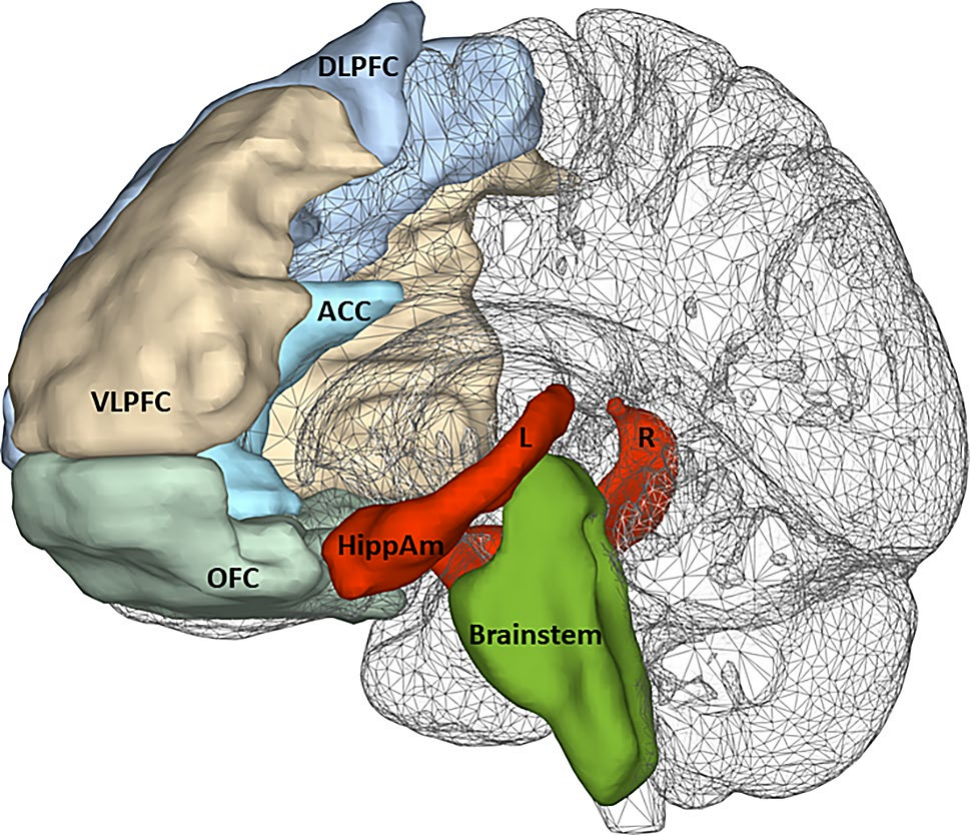
Sagittal glass brain view depicting the predefined volumes of interest (VOIs). The selected VOIS include brainstem, the dorsolateral prefrontal cortex (DLPFC), the ventrolateral prefrontal cortex (VLPFC), the orbital prefrontal cortex (OFC), the anterior cingulate cortex (ACC), and the hippocampal–amygdala complex (HippAM)

To test our hypothesis on 5-HT_2A_ receptor BI differences between depressed patients with and without comorbid GAD, first, in a MANCOVA analysis the six VOIs were analyzed as multiple dependent variables and MRD group (GAD versus no GAD) was the fixed between subjects’ factor. Given that age-dependent reductions in 5-HT_2A_ receptor-binding indices have consistently been reported [33, 34], we added age as covariate. Significant outcomes of the omnibus MANCOVA were followed up by univariate analyses. Second, to control whether 5-HT_2A_ receptor BI in these significant VOIs could be influenced by the individual daily dose intake of benzodiazepines (mg/day), the duration of the current depressive episode (years), suicide risk (MINI), or the individual depression severity scores (BDI-II), we performed an exploratory (partial) correlation analysis (corrected for age) for the entire sample and for each MRD group (GAD versus no GAD) separately. Third, to evaluate whether one of the eight symptom dimensions of the Dutch version of the SCL-90-R was related to any of the significant VOI 5-HT_2A_ receptor BI, again we performed an (partial) correlation analysis (corrected for age) between the significant VOIs and the eight SCL-90-R subscales for each MRD group separately (GAD versus no GAD), Bonferroni corrected. See Fig. 1.

## Results

### Demographics

MRD patients with (*n* = 10) or without GAD (*n* = 10) were not significantly different in age, gender distribution, benzodiazepine use, depression severity, or current episode duration. See Table 1. Of note, for one patient (in the MRD group with comorbid GAD), the SCL-90-R questionnaire went missing, so to evaluate the effects of SCL-90-R subscales only 19 patients were included. Here, we found that MRD patients with GAD scored significantly higher on insufficiency in thought and behavior (IN: *p* < 0.009) and nearly reached significance on Hostility (HOS; *p* = 0.052).

### Brain imaging

First, the MANCOVA analysis, with the different cortical VOIs (DLPFC, VMPFC, OFC, ACC, HippAM, and brainstem) as dependent variables, MRD group (GAD versus no GAD) as the between group fixed factor, and age as covariate, revealed a significant main effect of group [Wilks’ Lambda: *F*(6,12) = 3.48, *p* = 0.03, *η*_*p*_^2^ = 0.64], and age [Wilks’ Lambda: *F*(6,12) = 3.03, *p* = 0.048, *η*_*p*_^2^ = 0.60]. The individual univariate tests showed no significant age influences on 5-HT_2A_ receptor BI, although only for the hippocampal–amygdala complex this nearly reached significance [*F*(1,17) = 4.35, *p* = 0.052, *η*_*p*_^2^ = 0.20]. The univariate analysis for the other VOIs were not significant (*p*’s > 0.05). Furthermore, only for the hippocampal–amygdala complex, the individual univariate analysis showed a significant MRD group difference with a significant higher the 5-HT_2A_ receptor BI in patients with comorbid GAD (mean = 301.48, sd = 136.64) compared to those without comorbid GAD (mean = 232.62, sd = 68.80) [*F*(1,17) = 5.08, *p* = 0.038, *η*_*p*_^2^ = 0.23]. The other univariate analysis evaluating the other VOIs were not significant (*p*’s > 0.05). See also Table 2.

**Table 2:** VOI 5-HT_2A_ receptor BI

5-HT_2A_ receptor BI	All MRD patients	Without GAD	With GAD	F test	p value
VOIs					
DLPFC	104.50 (6.50)	104.69 (7.15)	104.32 (6.17)	0.05	0.83
VLPFC	107.71 (4.31)	108.05 (4.56)	107.38 (4.27)	0.34	0.57
OFC	107.85 (10.32)	107.33 (9.63)	108.38 (11.47)	0.35	0.56
ACC	106.68 (5.97)	107.35 (5.74)	106.01 (6.42)	< 0.01	0.97
HippAM	267.05 (111.05)	232.62 (68.80)	301.48 (136.64)	5.08	0.038
Brainstem	125.26 (16.27)	126.23 (17.98)	124.29 (15.27)	0.02	0.88

Univariate analysis

*VOIs* Volumes of interest, *DLPFC* the brainstem, the dorsolateral prefrontal cortex, *VLPFC* ventrolateral prefrontal cortex, *OFC* orbital prefrontal cortex, *ACC* anterior cingulate cortex, *HippAM* hippocampal–amygdala complex

All *p* values are age corrected

Second, partial correlation analysis (controlled for age) showed no significant association between HippAM 5-HT_2A_ receptor BI and the use of benzodiazepines, depression severity, suicide risk, and the duration of the current depressive episode for the entire sample, as well for the two groups (GAD vs. no GAD) separately (*p*’s > 0.01).

**Fig. 2:**
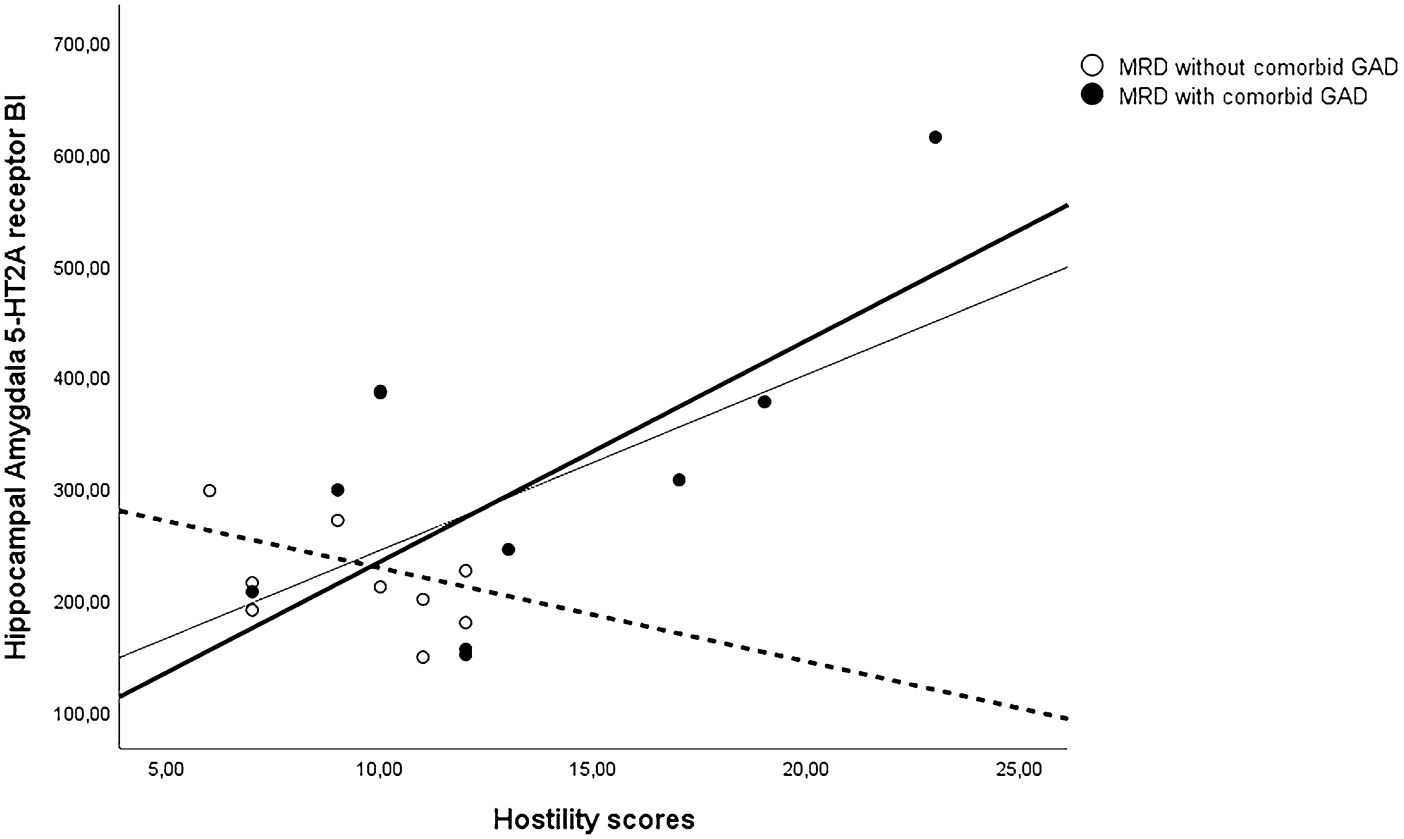
Scatter plots between the individual scores on hostility (HOS) (*x*-axis) and Hippocampal amygdala 5-HT_2A_ receptor BI complex (*y*-axis) for the entire medication-resistant depressed (MRD) sample (straight thin full line), and plotted separately for MRD patients with generalized anxiety disorder (GAD) (thick full line and full bullets) and without comorbid GAD (dashed thick line and open bullets). Of note, the lines representing the least-squares fit to the data are not corrected for age

Third, because group differences were found to be significant only for the HippAM VOI, for the (partial) correlation analysis with the SCL-90-R subscales, here, the *p* value was set at *p* < 0.00625, two-tailed (Bonferroni correction for eight SCL-90-R subscales). To evaluate whether any SCL-90-R questionnaire scales were associated with HippAM 5-HT_2A_ receptor BI (mean = 266.82, sd = 114.09), the partial correlation analysis (controlled for age: mean = 45.74, sd = 9.78) including all MRD patients showed a nearly significant positive correlation with the SCL-90-R subscale HOS only [(mean = 11.42, sd = 4.30), *r*(16) = 0.64, *p* = 0.007]. The results of the zero order correlation showed that there was a significant positive correlation between the HippAM 5-HT_2A_ receptor BI and hostility (HOS) only, *r*(17) = 0.59, *p* = 0.004, indicating that controlling for age had little effect on the strength of the relationship between the two variables. Separate explorative correlation analyses (not Bonferroni corrected) were separately performed in MRD patients with GAD and without GAD to further investigate whether the HippAM 5-HT_2A_ receptor BI and HOS correlation were any different between the two groups. Pearson partial correlation analysis showed that there was no significant correlation between both variables [*r*(8) = − 0.27, *p* = 0.23] in MRD patients without comorbid GAD. However, in MRD patients diagnosed with comorbid GAD, Pearson partial correlation analysis revealed a positive correlation between HippAM 5-HT_2A_ receptor BI and the individual HOS scores [*r*(7) = 0.71, *p* = 0.02]. See also Fig. 2. In addition, to assess the significance of the difference between the two correlation coefficients using the Fisher *r*-to-*z* transformation, which showed a significant difference (*z* = − 2.18, *p* = 0.03).

Finally, given the relatively small sample size and the potential influence of age differences between the two groups, as an additional manipulation check, we removed the oldest patient (female, 61 years, no GAD), and repeated all analyses. Besides that indeed there were no significant age differences between the two groups [*t*(17) = 1.52, *p* = 0.15], this did not affect any of the former outcome results, indicating that age was not a major influencer between the group analyses.

## Discussion

MRD patients with a comorbid diagnosis of GAD scored higher on insufficiency in thought and behavior (IN), and on hostility (HOS). Both behaviors have been reported when clinically depressed and/or when diagnosed with GAD [3, 15]. Whereas the IN dimension focuses on feelings of personal inadequacy and inferiority in comparisons with others—including self-deprecation, uneasiness, and discomfort during interpersonal interactions—higher scores on HOS indicates thoughts, feelings, or actions characteristic of the negative affect state of anger reflecting qualities such as aggression, irritability, rage and resentment [29]. Besides that, the demographics in both groups were similar (see Table 1), depression and anxiety SCL-90-R subscale scores did also not differ between the two groups, which suggests that the levels of depression and anxiety symptoms were not influenced by the occurrence of comorbid GAD in our MRD sample.

Concerning our SPECT results, the lack of frontal cortical group 5-HT_2A_ receptor BI differences in our sample indicates that serotonergic influences may not differ in MRD with or without comorbid GAD, suggesting no top–down cognitive different influences from the frontal cortical areas [5]. Of note, findings concerning the frontal cortical 5-HT_2A_ receptor in the depressed state are not univocal, with some authors demonstrating 5-HT_2A_ receptor increases, and others demonstrating decreases, or no differences in receptor ligand binding at all [28]. On the other hand, the observed increased (hippocampal) amygdala 5-HT_2A_ receptor BI agrees with its modulating capacity of anxiety-states and anxiety-related behavior and are probably modulated by GABAergic interneurons in the (basolateral) amygdalae [35]. Of interest, in patients with Urbach–Wiethe disease, displaying severe bilateral amygdala damage, this syndrome has been associated with decreases in amygdala 5-HT_2A_ receptor density and related to decreased fear and anxiety behavioral manifestations [36]. In addition, because of its involvement in the hypothalamic–pituitary–adrenal (HPA)-stress system, the role of the hippocampus in the glucocorticoid regulation of stress reactions is well known [37]. Here, the HPA-system is thought to be regulated by serotonin in hippocampal neurons [38, 39], and especially by hippocampal 5-HT_2A_ receptor activation [40].

It is of interest to note that in patients suffering from Borderline Personality Disorder—an emotional unstable and stress-sensitive personality disorder characterized amongst other symptoms with separation anxiety, anger and impulse control disturbances—only hippocampal 5-HT_2A_ receptor binding was increased, independent of depressed mood [41]. Our (partial) correlation analysis showed that increases in HippAM 5-HT_2A_ receptor BI were related to higher scores on the SCL-90-R subscale score hostility (HOS) in the MRD state. Common with BPS symptoms, our exploratory partial correlation analysis also showed that increased HippAM 5-HT_2A_ receptor BI in relation to increased hostility could me especially apparent in MRD patients with comorbid GAD. In addition, GAD may lead to emotional dysregulation, including unsuppressed anger and low tolerance to frustration [42]. In addition, higher levels of anger and hostility contributed and predicted GAD symptom severity and elevated levels of multiple dimensions of anger characterized individuals who met the diagnostic criteria for GAD [16].

In preclinical studies, the involvement of the 5-HT_2A_ receptor in anxious behavior has been observed in dogs [43], and disinhibited aggressive behavior associates with an increased cortical uptake of the 5-HT_2A_ receptor radioligand ^123^I-5-IR91150 [44]. Of interest, successful SSRI treatment in impulsive-aggressive dogs downregulated the cortical 5-HT_2A_ receptor BI also in the temporal cortical areas, in canine species regarded as part of the hippocampal complex [45]. In depressed adolescents, a single dose of fluoxetine also immediately reduced neural amygdala–hippocampal activity in response to angry facial expressions [46]. Furthermore, among outpatients with MDD early clinical improvement in anger/hostility symptoms may predict response to antidepressant treatment with fluoxetine [47]. It may of interest to note that, although we focused on 5-HT_2A_ receptor BI in the frontolimbic regions, with inclusion of the brainstem, the investigated VOIs are structurally connected to the medial forebrain bundle, primarily mediated by the dopaminergic system as being part of the reward pathway targeting similar brain regions also selected for our current 5-HT_2A_ post-synaptic receptor study (MFB; [48]). In TRD patients, these (superolateral) MFB has been recently the target areas for deep-brain stimulation (DBS; [49, 50]) offering depressed patients unresponsive to several pharmacotherapeutic interventions an option to relief their mental burden. It would be of interest to verify in future DBS studies in depression whether clinical improvement also differentially attenuates hostility thoughts and actions in TRD patients diagnosed with or without GAD.

Patients diagnosed with GAD tend to avoid intense negative emotions by engaging in worry or behavioral avoidance, which may lead to an increase in emotional distress [51], displaying an elevated intolerance of uncertainty (IU) and anger [52]. Importantly, individual and group psychotherapeutic sessions targeting this IU construct in GAD patients may result not only in decreases in IU, but also on worrying and on angriness [18, 53]. One could speculate that MRD patients with comorbid GAD may benefit from such specific additional psychotherapeutic interventions, impacting elevated HippAM 5-HT_2A_ receptor BI.

Besides the obvious limitation, the relatively small sample size, all interpretations should be limited to melancholic MRD patients. Since we have not contrasted our findings to a non-depressed group, a GAD group with no comorbid (treatment resistant) depressed episode, the absence of cortical group 5-HT_2A_ receptor BI only implies that in the MRD cohort frontal 5-HT_2A_ receptor densities are not significantly different with co-occurrence of GAD, and that the hippocampal–amygdala serotonergic findings are specific for the comorbid existence of MRD and GAD. A healthy control group could also have been informative to examine whether 5-HT_2A_ receptor VOI-binding indices would be any different between the healthy and the depressed state, and to evaluate whether in healthy samples hostility symptoms would also be related to higher HippAM 5-HT_2A_ receptor densities. Although MDD was the primary diagnosis, it remains possible that for some the diagnoses of GAD preceded the MDD episode, and not vice versa (e.g., [54]). We used the MINI as a diagnostic tool, but the severity of GAD symptoms was not examined with disease specific questionnaires. The MINI does also not include any personality diagnostic measurement. Although the amygdala and hippocampus may serve different neuronal and behavioral functions, the limited resolution of the SPECT scan prohibited clear disentanglement of 5-HT_2A_ receptor BI between the two anatomical structures [55]. Future PET ligand studies, more capable to delineate between hippocampus and amygdala could examine potential 5-HT_2A_ receptor BI regional differences. Of note, given the sample size of only 20 MRD patients, due to potential insufficient statistical power, the negative findings of the other VOIs may be a false negative. Since left and right VOIs were pooled due to the relatively small sample size, we cannot comment on possible lateralization influences.

In conclusion, the increased hippocampal–amygdala 5-HT_2A_ receptor BI found in depressed patients documented to be resistant to several antidepressant interventions and with the comorbid diagnosis of generalized anxiety could represent a serotonergic biomarker for this comorbid occurrence. Furthermore, the enhanced levels in hostility related to hippocampal–amygdala 5-HT_2A_ receptor BI in such kinds of patients displaying this comorbidity could indicate that adapted psychotherapeutic interventions may be warranted. It remains without saying that these preliminary findings should be replicated in larger clinical samples to substantiate our assumptions.

## Data Availability

(Data transparency). Data will be made available on reasonable request.

## Code availability

(Software application or custom code). Data will be made available on reasonable request.
